# Supplementary material for: Manipulation of Alternative Splicing of IKZF1 Elicits Distinct Gene Regulatory Responses in T Cells
Source: Cells. 2026 Jan 24;15(3):221. doi: 10.3390/cells15030221 (PMC12896614; doi:10.3390/cells15030221)
Supplement: Supplementary file 1 [file cells-15-00221-s001.zip › cells-3960050-supplementary.pdf]

Supportive Information

Supplemental Note

A Figshare repository has been created (DOI reference: 10.6084/m9.figshare.24878328) to deposit data pertaining to the analyses presented in this study. The repository contains the raw data presented in Figures 3B, 4, 5, and 7 of the main manuscript and Figures S2, S3, and S4.

The repository also contains the complete and annotated results tables for differential gene expression and chromatin accessibility analyses to test the hypotheses that changes in gene transcription and chromatin structure related to *IKZF1* would be the following:

1. Dependent solely on whether *IKZF1* was wild-type (WT) or exon-targeted (column "Edited vs WT" in **Table S2**);
2. Dependent on the specific exon or exons targeted (column "By exon edited" in **Table S2**);
3. Dependent on the specific exon(s) targeted and detectable IKZF1 protein expression (column "By exon edited and protein detection" in **Table S2**);
4. Dependent on the number of exons targeted by CRISPR (column "By number of exons edited" in **Table S2**).

These data are presented for the benefit of readers who wish to query the impact of *IKZF1*-targeting on the expression or chromatin state of specific genes. The raw sequence data used to generate these results are available as FASTQ files from NCBI's Gene Expression Omnibus accessions GSE252707 and GSE255337.

Table S1: Characteristics of Jurkat clones derived from CRISPR editing of *IKZF1*

Clone	Exon target	sgRNA	Sequence variant [a]	Genomic coordinate [b]
C1	WT	n/a	c.=	chr7:g.=
C2	WT	n/a	c.=	chr7:g.=
C3	WT	n/a	c.=	chr7:g.=
C4	WT	n/a	c.=	chr7:g.=
C5	4	4sg1	c.220_221ins[GG;CT]	chr7:g.50376592_50376593ins[GG;CT]
C6	4	4sg1	c.220_221ins[C;GT]	chr7:g.50376592_50376593ins[C;GT]
C7	4	4sg1	c.220_221insCC	chr7:g.50376592_50376593insCC
C8	4	4sg1	c.223delG	chr7:g.50376598delG
C9	4	4sg1	c.220_221ins[CC;GT]	chr7:g.50376592_50376593ins[CC;GT]
C10	4	4sg2	c.260_261ins[G;T]	chr7:g.50376632_50376633ins[G;T]
C11	4	4sg2	c.260_261insT	chr7:g.50376632_50376633insT
C12	6	6sg1	c.685_686ins[GGGG;CCCT]	chr7:g.50387440_50387449ins[GGGG;CCCT]
C13	6	6sg1	c.685_686ins[C;GGAA]	chr7:g.50387440_50387449ins[C;GGAA]
C14	6	6sg2	c.694_695insC	chr7:g.50387449_50387450insC
C15	6	6sg2	c.694_695insA	chr7:g.50387449_50387450insA
C16	6	6sg2	c.694delA	chr7:g.50387449delA
C17	4+6	6sg2 + 4sg1	c.220_221ins[G;GCCC];694delA	chr7:g.50376592_50376593ins[G;GCCC];50387449delA
C18	4+6	6sg2 + 4sg1	c.220_221insG;694delA	chr7:g.50376592_50376593insG;50387449delA
C19	4+6	6sg2 + 4sg1	c.220_221ins[GG;GT];694delA	chr7:g.50376592_50376593ins[GG;GT];50387449delA
C20	4+6	6sg2 + 4sg1	c.220_221[insT;delinsCCC];694delA	chr7:g.50376592_50376593ins[insT;delinsCCC];<br>50387449delA
C21	4+6	6sg2 + 4sg1	c.[221_222insG;694delA];<br>[218_222delinsA;694delA]	chr7:g.[50376592_50376593insG;50387449delA];<br>[50376590_50376594delinsA;50387449delA]
C22	4+6	6sg2 + 4sg1	c.220_221ins[GCTA;GGACC];694delA	chr7:g.50376592_50376593ins[GCTA;GGACC];<br>50387449delA
C23	4+6	6sg2 + 4sg1	c.220_221insC;694delA	chr7:g.50376592_50376593insC;50387449delA
C24	4+6	6sg2 + 4sg1	c.220_221insGGTC; 694delA	chr7:g.50376592_50376593insGGTC;50387449delA

[a] Reference sequence ENST00000331340.8; [b] Reference genome GRCh38.p13 (chromosome accession NC_000007.14); WT—wild type.

Table S2: Group membership of Jurkat clones

Clone	Targeted vs WT	By exon targeted	By exon edited and protein detection (Figure 2)	By number of exons edited	Exon 4 allele sequences	Exon 6 allele sequences
1	WT	WT	WT	WT	WT	WT
2	WT	WT	WT	WT	WT	WT
3	WT	WT	WT	WT	WT	WT
4	WT	WT	WT	WT	WT	WT
5	Targeted	4	4, low protein	Single	GA**ins[GG;CT]**ATGGGG	WT
6	Targeted	4	4, low protein	Single	GA**ins[C;GT]**ATGGGG	WT
7	Targeted	4	4, low protein	Single	GA**ins[CC]**ATGGGG	WT
8	Targeted	4	4, high protein	Single	GAAT**del[G]**GGG	WT
9	Targeted	4	4, low protein	Single	GA**ins[CC;GT]**ATGGGG	WT
10	Targeted	4	4, high protein	Single	GC**ins[G;T]**CTCGGG	WT
11	Targeted	4	4, high protein	Single	GC**ins[T]**CTCGGG	WT
12	Targeted	6	6	Single	WT	AA**ins[GGGG;CCCT]**GCATGGG
13	Targeted	6	6	Single	WT	AA**ins[C;GGAA]**GCATGGG
14	Targeted	6	6	Single	WT	CC**ins[C]**TTCCGG
15	Targeted	6	6	Single	WT	CC**ins[A]**TTCCGG
16	Targeted	(excluded)	(excluded)	(excluded)	WT	C**del[C]**TTCCGG
17	Targeted	6+4	6+4	Double	GA**ins[G;GCCC]**ATGGGG	C**del[C]**TTCCGG
18	Targeted	6+4	6+4	Double	GA**ins[G]**ATGGGG	C**del[C]**TTCCGG
19	Targeted	6+4	6+4	Double	GA**ins[GG;GT]**ATGGGG	C**del[C]**TTCCGG
20	Targeted	6+4	6+4	Double	GA**[ins[T]A;ins[CCC]del[A]]**TGGGG	C**del[C]**TTCCGG
21	Targeted	6+4	6+4	Double	GAA**[insG;del[TGAAT]ins[A]]**GGGG	C**del[C]**TTCCGG
22	Targeted	6+4	6+4	Double	GA**ins[GCTA;GGACC]**ATGGGG	C**del[C]**TTCCGG
23	Targeted	6+4	6+4	Double	GA**ins[C]**ATGGGG	C**del[C]**TTCCGG
24	Targeted	6+4	6+4	Double	GA**ins[GGTC]**ATGGGG	C**del[C]**TTCCGG

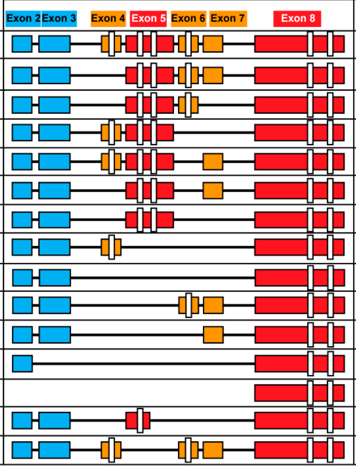

Isoform	Length	kDa	Uniprot ID	Ensembl ID	Genbank	Isoform structure	Potential expression WT	EX4	EX6	EX4+6	
Ik-1	519 aa	57.54	Q13422-1	ENSP00000331614	NP_006051		+				DNA binding isoforms
Ik-2	432 aa	48.30	Q13422-2	ENSP00000342750	NP_001278767		+	+			
Ik-2a	387 aa	43.22					+	+			
Ik-3	432 aa	47.64	Q13422-3	ENSP00000349928	NP_001207697		+		+		
Ik-3a	477 aa	52.71	Q13422-7	ENSP00000352123	NP_001207694		+		+		
Ik-4	390 aa	43.47		ENSP00000342750	NP_001278768		+	+	+	+	
Ik-4a	345 aa	38.40					+	+	+	+	
Ik-5	376 aa	41.24	Q13422-5	ENSP00000342485	NP_001207700		+		+		Dominant-negative isoforms
Ik-6	289 aa	31.99	Q13422-6		NP_001278769		+	+	+	+	
Ik-7	376 aa	41.89			NP_001278770		+	+			
Ik-8	334 aa	37.07			NP_001278772		+	+	+	+	
Ik-9	239 aa	27.85		ENSP00000340080			+	+	+	+	
Ik-10	215 aa	24.18					+	+	+	+	
Ik-11	248 aa	27.67					+	+	+	+	
Ik-12	463 aa	51.13					+				

Figure S1: Characteristics of *IKZF1* isoforms and the impact of gene-editing events. Indicated are the lengths in amino acids (aa), molecular weights (kDa), database IDs, and structures of documented isoforms of *IKZF1*/Ikaros that include both the DNA-binding and dominant-negative isoforms. Potential expression indicates which Ikaros isoforms can be produced in Jurkat clones with editing events in exon 4, exon 6, or both. WT—wild-type; EX4—exon 4; EX6—exon 6; EX6+4—both exons. Exons alternatively spliced among the reported IKAROS functional isoforms are colored orange. Exons containing zinc fingers but that are not alternatively spliced are indicated in red. All other exons are colored blue.

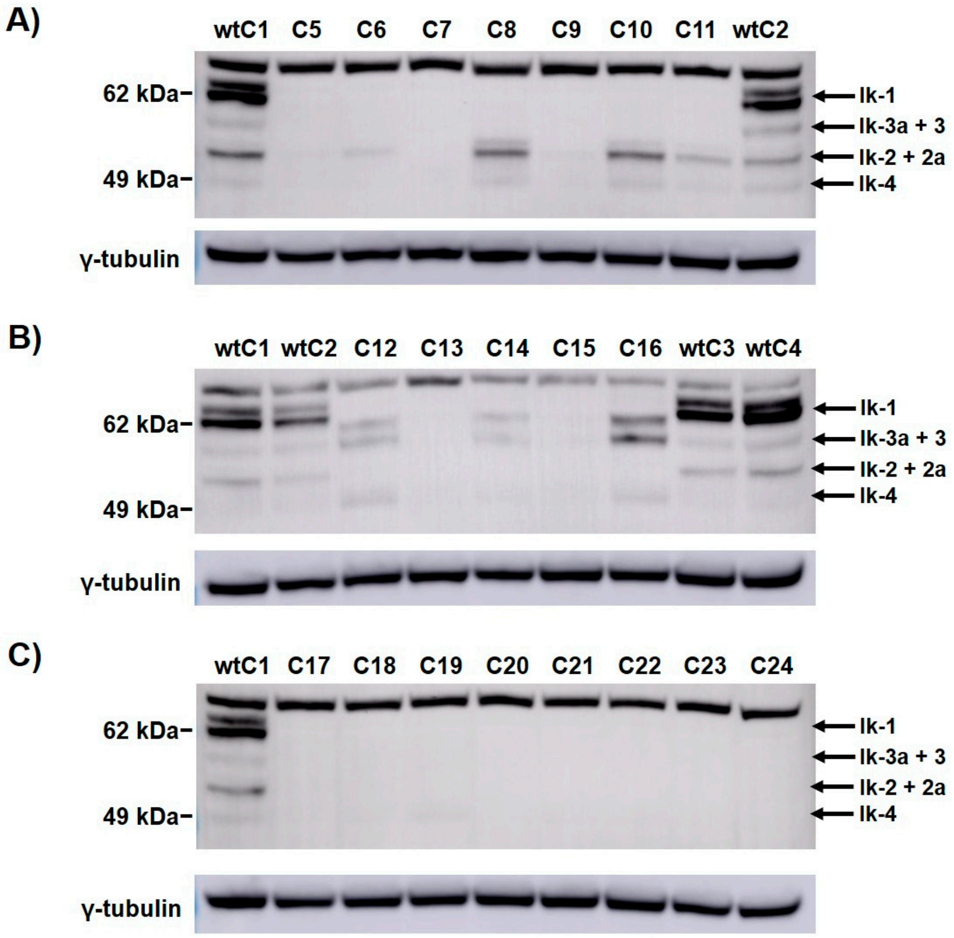

Figure S2: IKAROS protein expression in targeted clones. IKAROS protein expression as assayed by immunoblotting of clones with targeting events in exon 4 (A), exon 6 (B), and both exons (C), as compared to wild-type clones (WT), using a primary antibody specific for the C-terminal end of the protein. The same blots were stripped and probed with an antibody against γ-tubulin as a protein loading control. Black arrows indicate the expected sizes of IKAROS isoforms containing the N-terminal zinc fingers encoded by exon 5 (i.e., Ik-1, Ik-2, Ik-2a, Ik-3, Ik-3a, and Ik-4; **Figure S1**).

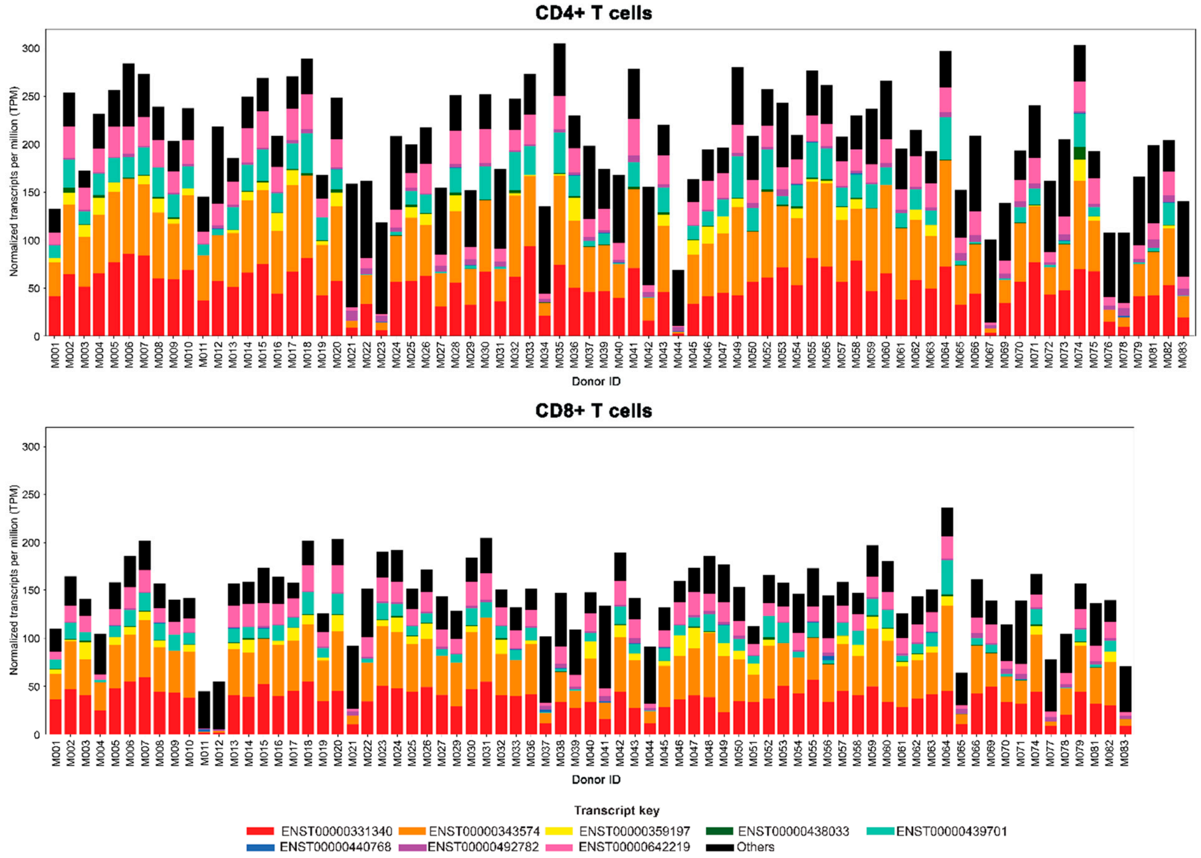

Figure S3: Distribution of IKZF1 transcript expression in primary CD4+ and CD8+ T-cell donors. The top-expressing transcripts in Jurkat cell lines are color-coded by transcript identifier, as in Figure 3, with their expression in TPM represented in the stacked bars for each donor. Total bar height corresponds to the total *IKZF1* gene expression for the indicated donor.

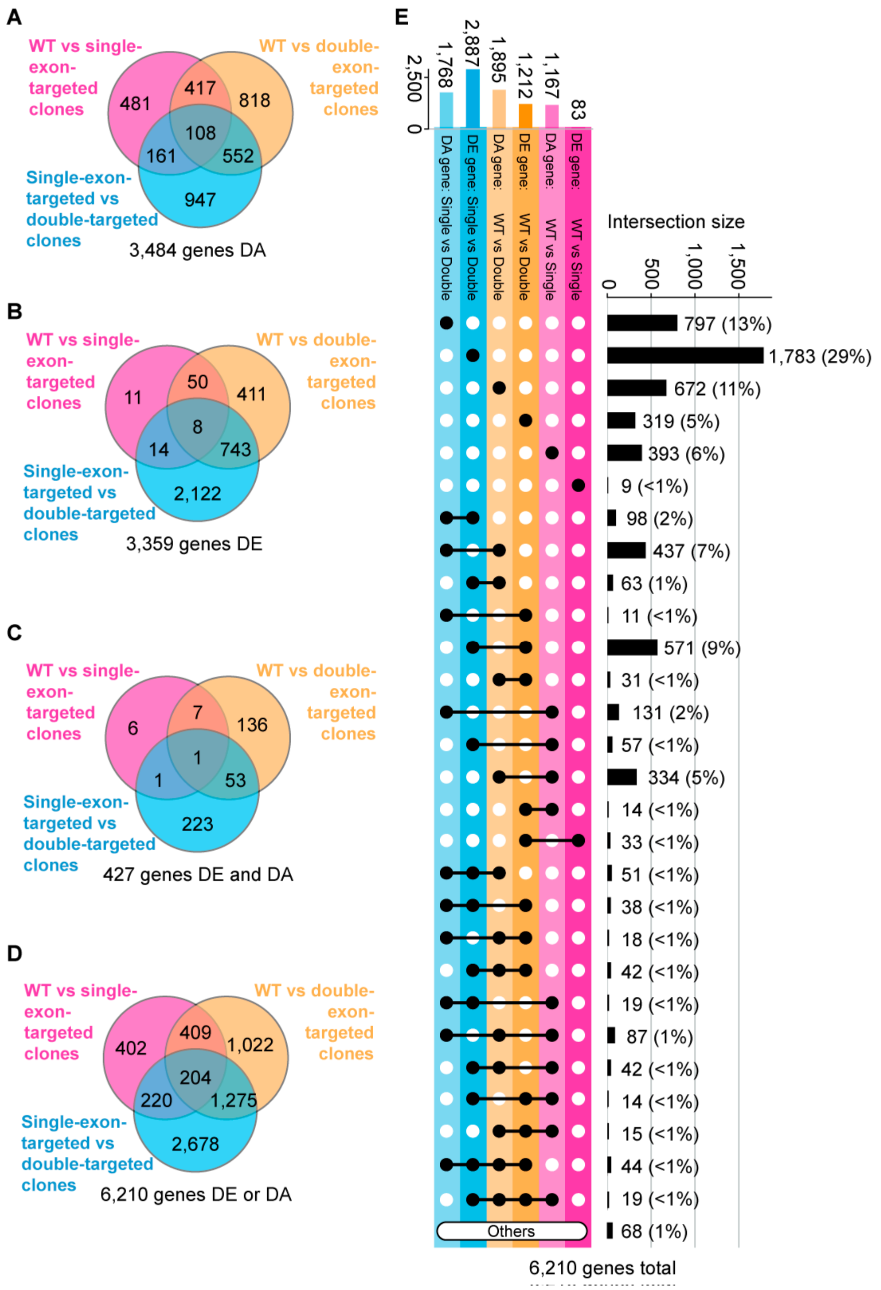

Figure S4: Comparison of differentially accessible (DA) and/or differentially expressed (DE) genes between WT, single-exon-targeted clones, and double-exon-targeted clones. (A) DA genes only, (B) DE genes only, (C) genes classed as both DA and DE, (D) genes classed as either DA or DE. (E) Upset plot comparing statistically significant genes between WT, single-targeted, and double-targeted clones for each assay (chromatin accessibility, gene expression). Intersections

with fewer than 10 members (excluding genes only DE between WT and single-exon-targeted clones) are grouped into the "Others" category.

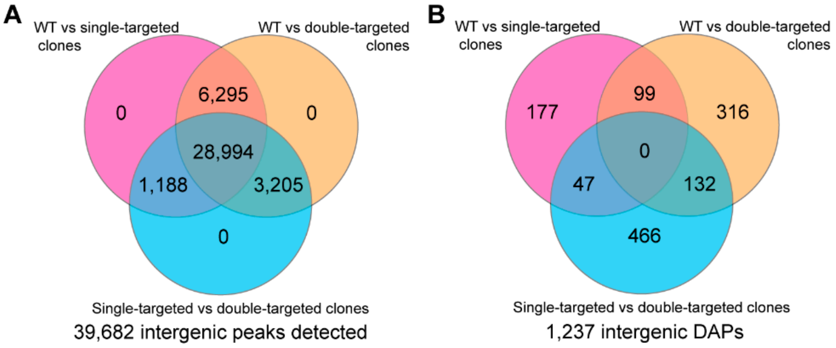

Figure S5: Differentially accessible ATAC-seq peaks in intergenic regions. (A) Intergenic ATAC-seq peak analyzed in each comparison made. (B) Overlap of all intergenic DAPs between WT, single-targeted, and double-targeted clones.

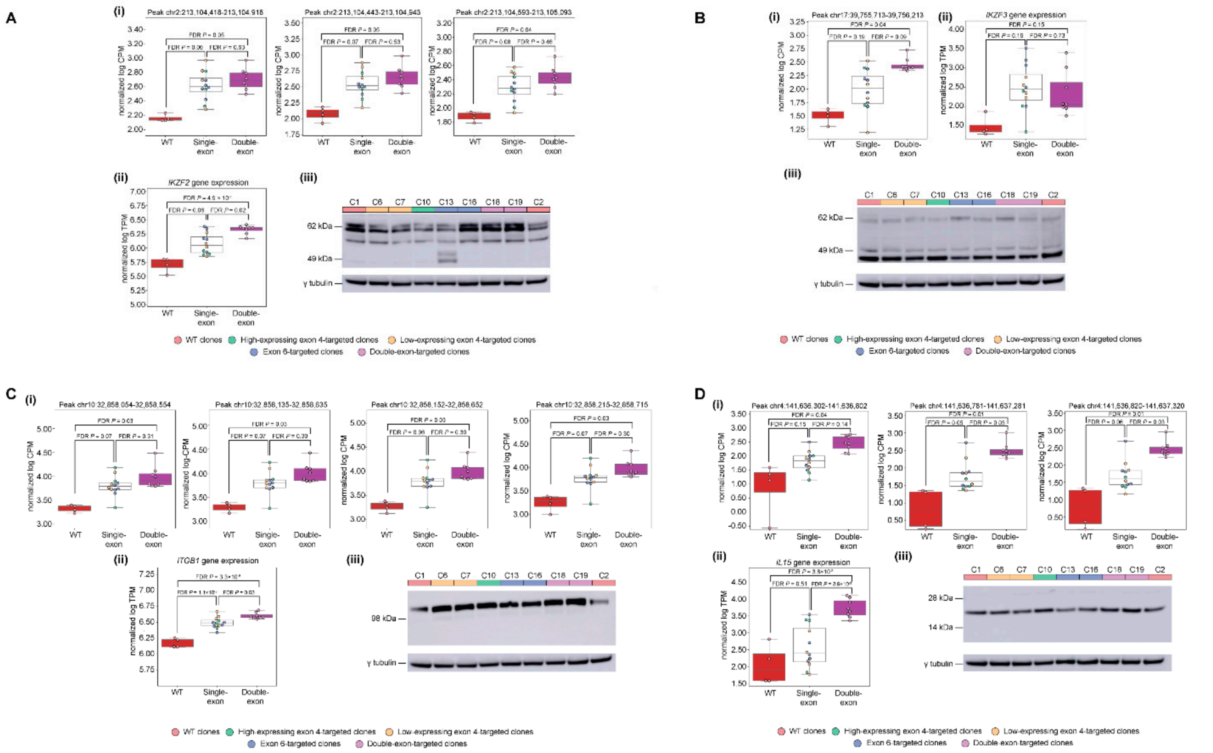

Figure S6: Effects of *IKZF1* editing on example genes, *IKZF2* (A), *IKZF3* (B), *ITGB1* (C), and *IL15* (D), in terms of chromatin accessibility (i), gene expression (ii), and protein levels (iii). All changes in chromatin accessibility associated with each gene are presented here. Red boxplots—WT clones; white boxplots—single-exon clones; purple boxplots—double-exon clones; pink/red dots—WT clones; green dots—low protein-expressing exon 4-targeted clones; yellow dots—high protein-expressing exon 4-targeted clones; blue dots—exon 6-targeted clones; purple dots—double-exon clones.
